# Clinical feasibility of intratracheal tracheostomy sealing using a novel sealing disc prototype

**DOI:** 10.1038/s41598-026-41209-8

**Published:** 2026-03-24

**Authors:** Rasmus Ellerup Kraghede, Louise Winding Nielsen, Karen Juelsgaard Christiansen, Stig Dyrskog, Nilanjan Dey, Alexander Emil Kaspersen, J. Michael Hasenkam

**Affiliations:** 1 Department of Intensive Care, Aarhus University Hospital, Aarhus, Denmark; 2 Department of Cardiothoracic and Vascular Surgery, Aarhus University Hospital, Aarhus, Denmark; 3 Department of Clinical Medicine, Faculty of Health, Aarhus University, Aarhus, Denmark; 4 Department of Anaesthesiology and Intensive Care, Regional Hospital Gødstrup, Herning, Denmark

**Keywords:** Diseases, Health care, Medical research

## Abstract

After tracheostomy decannulation, stoma openings compromise airway integrity, reduce subglottic pressure, and impair cough efficiency. Conventional external dressings are often not airtight and require extensive maintenance, increasing the risk of delayed healing and pulmonary complications. We evaluated the feasibility, physiological impact, and patient-reported outcomes of an intratracheal sealing disc designed to maintain airway integrity during early post-decannulation healing. In this feasibility study, 21 tracheostomized patients were enrolled from the Department of Intensive Care at Aarhus University Hospital and Gødstrup Regional Hospital (pilot phase, *n* = 11; main study, *n* = 10). Immediately after decannulation, a sealing disc was inserted intratracheally and secured externally for up to 7 days. Spirometry, voice quality, and tracheostomy healing were assessed daily. Patient satisfaction was measured using a questionnaire with a 5-point Likert scale. The sealing disc provided an airtight closure during speaking and coughing. Placement and removal were quick and uneventful. Patient satisfaction overall reached a median (interquartile range) score of 5/5 (1). Forced vital capacity, forced expiratory volume in the first second, and voice quality improved significantly immediately after sealing and remained stable during wear and after removal. In this First-in-Man clinical feasibility study, intratracheal sealing immediately after decannulation proved feasible, well-tolerated, and may improve pulmonary function, phonation, and wound healing. Larger controlled studies are warranted.

**Trial Registration**: This trial was retrospectively registered at ClinicalTrials.gov with the identifier NCT07149116 – date of registration: 08/08/2025.

**Supplementary Information:**

The online version contains supplementary material available at 10.1038/s41598-026-41209-8.

## Introduction

Tracheostomy is often used in intensive care for prolonged mechanical ventilation^1,2^. Tracheostomy decannulation – the process of removing a tracheostomy tube – leaves an open stoma in the airways that typically closes spontaneously within 7–14 days^3–5^. Meanwhile, the presence of the stoma compromises airway integrity and hinders the coughing mechanism and pulmonary rehabilitation^6–8^. Additionally, the open stoma can prevent the use of positive expiratory pressure (PEP) devices and continuous positive airway pressure (CPAP) therapy, thereby limiting effective lung recruitment and clearance of airway secretions^9–11^. Current post-decannulation care typically involves the application of occlusive dressings or gauze bandages; however, these methods are not airtight. Inadequate sealing of the tracheostomy site hinders effective coughing, thereby increasing the risk of decannulation failure and prolonging patient recovery^8,12^. Furthermore, air leakage through the tracheostoma reduces the patient’s voice quality and is associated with inadequate patient care and social isolation^13,14^.

Previous studies have explored an innovative approach to address these challenges^15,16^. The approach involves intraluminal sealing of the stoma by deploying a silicone-based disc through the tracheostoma to the inside of the trachea, thereby restoring subglottic pressure and enabling effective coughing, which supports alveolar recruitment, secretion clearance, and overall respiratory recovery^10,16–19^. In an acute clinical feasibility study, we demonstrated that this intratracheal tracheostomy sealing improved lung function in addition to enhancing the patient’s voice quality, immediately after decannulation^16^. These findings suggest that effective sealing of the tracheostomy can significantly benefit respiratory physiology after tracheostomy decannulation. A further developed prototype of this approach was investigated in an animal model^18^. It created an airtight seal of the tracheostomy despite pressurization of the airways, whilst not promoting excessive inflammation in the tissue or causing airway obstruction. The tested sealing disc was designed to be secured by a cord that passed through the stoma to an external component, allowing for easy removal through the skin once wound healing was complete.

Building on these initial findings, we developed a third-generation intratracheal sealing disc designed to provide immediate, airtight closure of the tracheostomy after decannulation. We hypothesize that such sealing may preserve airway pressure, facilitate effective coughing, enhance pulmonary rehabilitation, and accelerate wound healing. The primary aim of this study was to evaluate the safety, feasibility, and clinical acceptability of this sealing disc prototype in a post-decannulation setting. Secondary objectives include assessing changes in respiratory physiology and voice quality during the early phase of tracheostomy wound healing. Through this investigation, we aim to generate proof of concept for a future approach to post-decannulation treatment of tracheostomized patients after prolonged mechanical ventilation.

## Methods

This feasibility study addresses the performance and safety of a novel intratracheal sealing disc prototype in a cohort of ten adult patients following prolonged mechanical ventilation via tracheostomy. Feasibility was defined a priori as successful device deployment and use without device-related adverse events or need for reintervention. The study time window was up to seven days from tracheostomy decannulation until healing of the tracheostomy, where the sealing disc was removed. Prior to the main study, a pilot phase involving eleven patients was conducted to refine the deployment technique and optimize data collection procedures.

### The tracheostomy sealing disc prototype

**Fig. 1:**
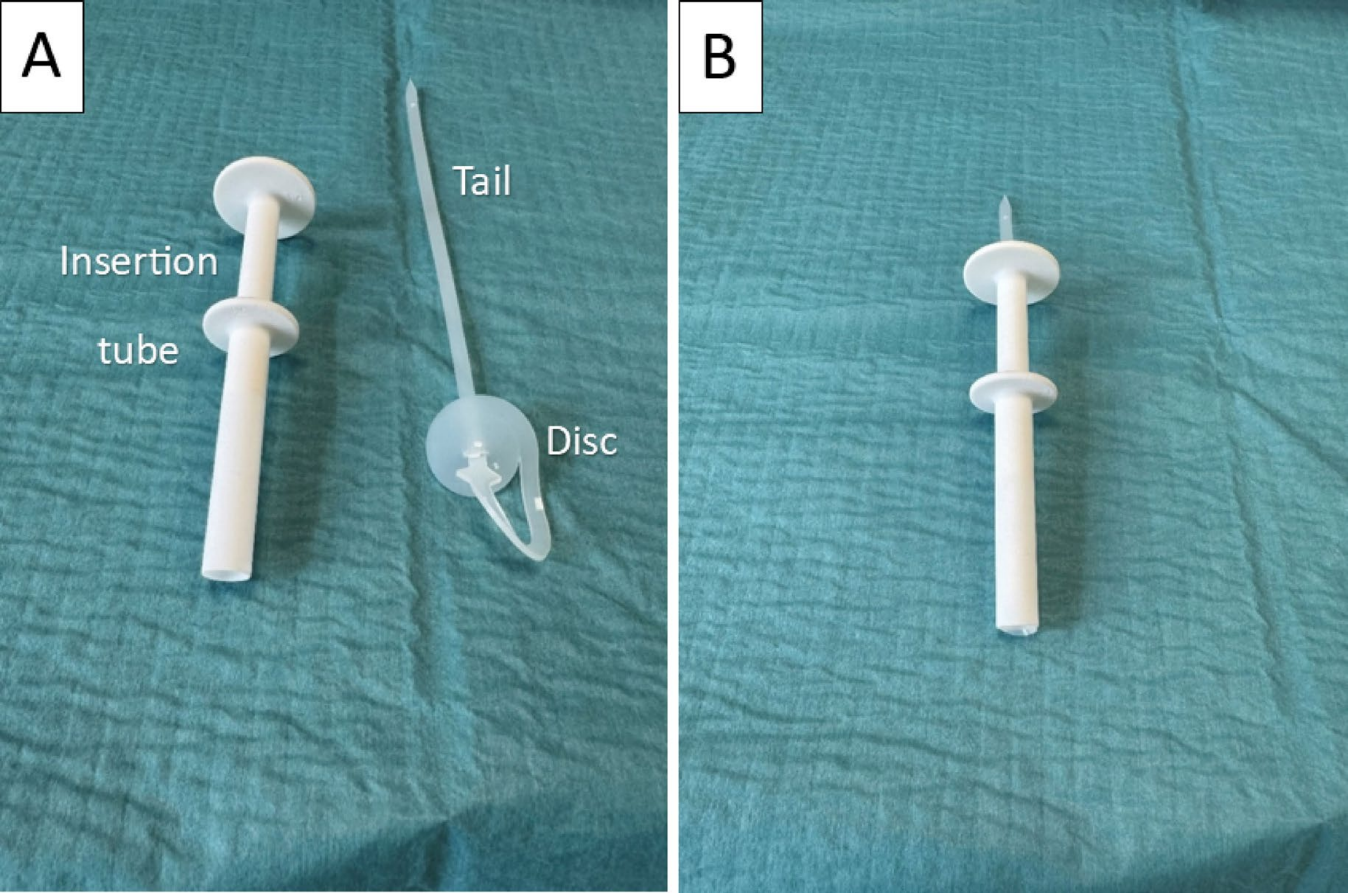
The third-generation tracheostomy sealing disc and the insertion tube (**A**) separated and (**B**) ready for insertion with the sealing disc placed in the insertion tube.

**Fig. 2:**
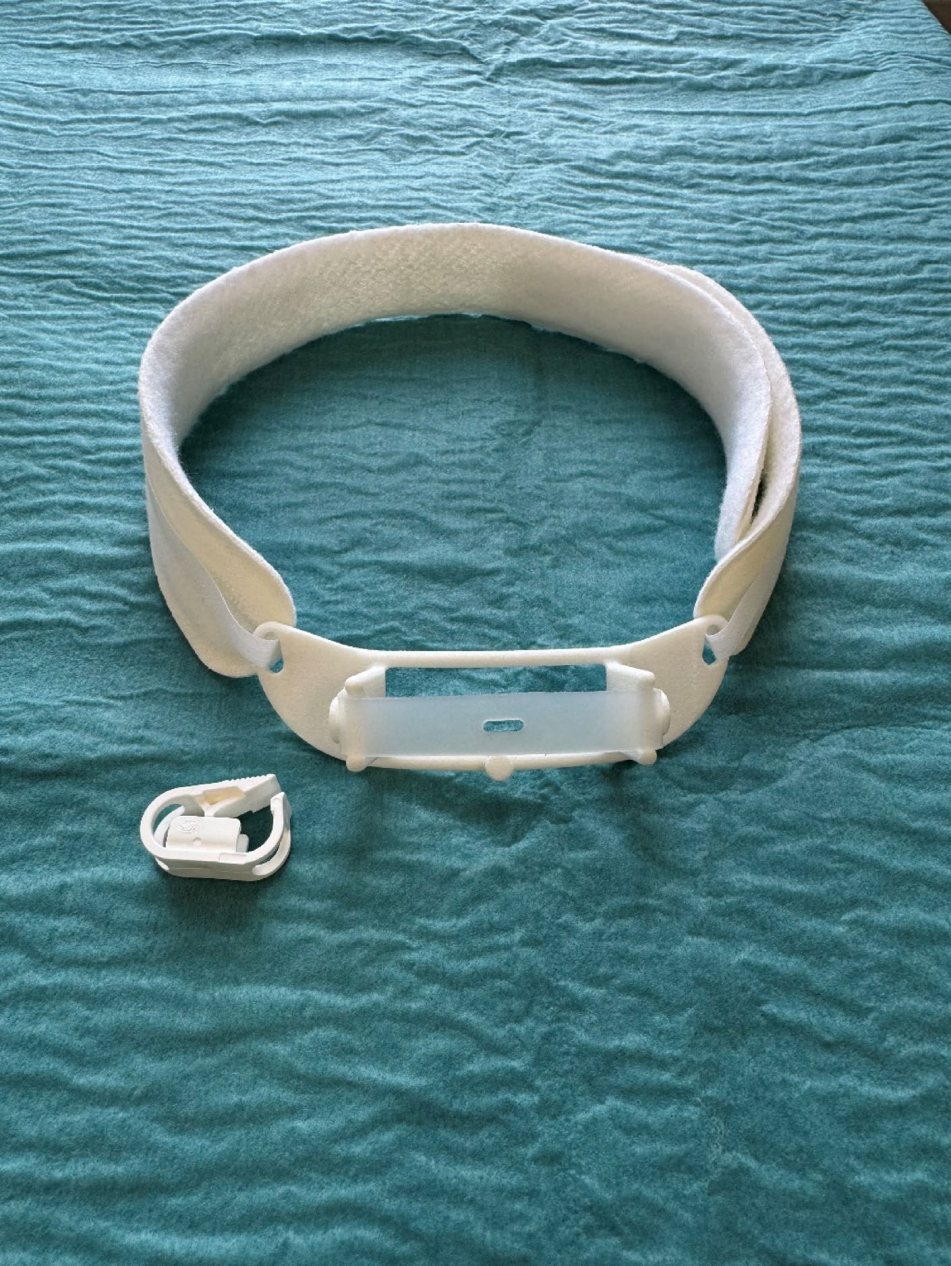
External fixation system of the tracheostomy sealing disc.

The sealing disc, also described in a previous study^18^, consists of a single strand of silicone spiraled together to form a circular disc on one end and a long “tail” on the other. The disc measures 30 mm in diameter with a thickness of 2 mm. The “tail” is 5 mm wide and 2 mm thick. The sealing disc is able to airtightly seal the tracheostomy from the intraluminal site while being fixated externally on the front of the neck. The sealing disc is designed to be deployed through the tracheostomy channel using a specially engineered insertion tube (Fig. 1). The sealing disc is held in place with a white tube clamp from a feeding tube (Fresenius Kabi AG, 61346 Bad Hamburg, Germany) that rests on a suspended piece of silicone acting as a dampener on the external fixation (Fig. 2). The bulge seen at the junction between the disc and the “tail” represents an intentional safety feature that increases the force required to initiate device removal. In combination with the external silicone bridge, this allows for constant tension on the sealing disc and avoids unintentional removal during patient movement and coughing. At the tail end of the sealing disc, a circular hole has been added to secure the sealing disc to a button on the external fixation system if the clamp breaks.

### Sealing disc deployment

**Fig. 3:**
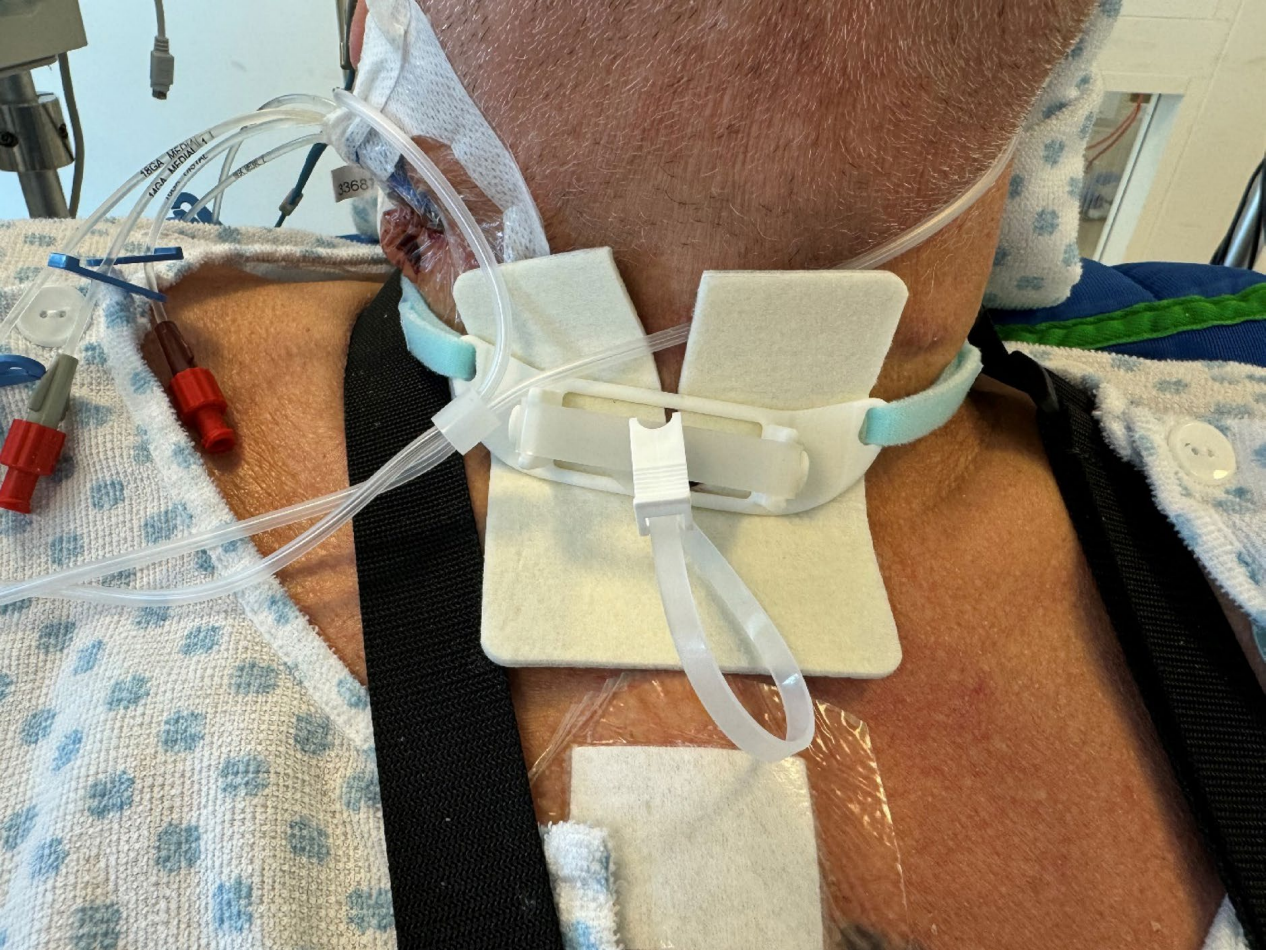
A patient with the tracheostomy sealing disc inserted and fixated to the external fixation system. Published with permission of the patient.

Before insertion, the sealing disc is delicately folded and placed in the insertion tube to ensure proper intratracheal positioning. Once deployed, the disc is stabilized by the external fixation system, which is secured to the patient’s neck using an adjustable neck band. The wound is covered with a gauze to manage wound secretion (Fig. 3). The process of inserting the sealing disc through the tracheostomy can be seen in Supplementary Video S1.

### Sealing disc removal

**Fig. 4:**
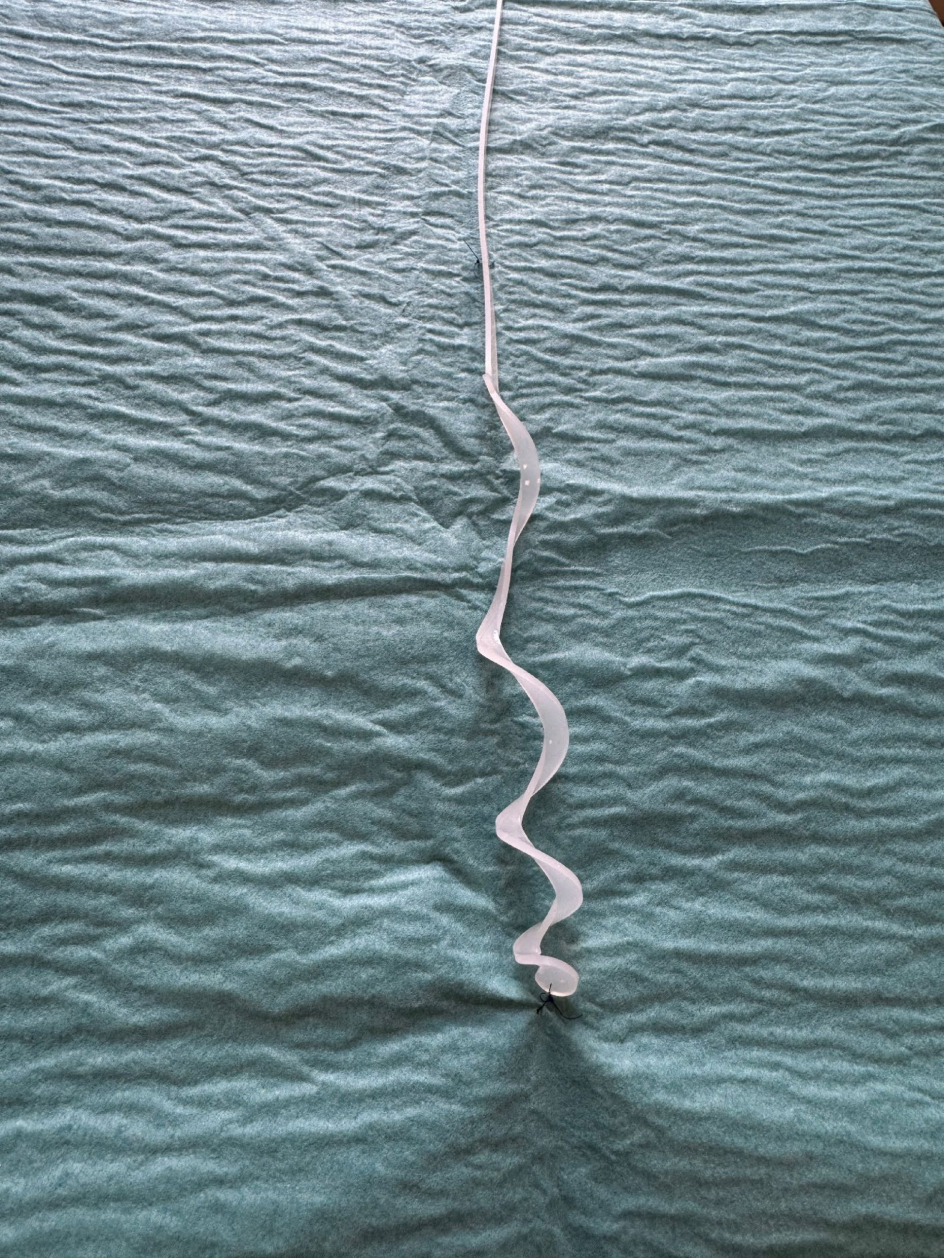
The tracheostomy sealing disc uncoiled.

The sealing disc is removed by applying gentle traction to the retrieval string, which causes the disc to uncoil inside the tracheal lumen. Once uncoiled, the sealing disc assumes a linear string configuration, allowing for atraumatic extraction through the narrow residual tracheostomy wound (Fig. 4). The wound is then covered with an occlusive dressing. Supplementary Video S2 shows a bronchoscopic recording of the uncoiling process in a preclinical porcine model, using a sealing disc prototype identical to the prototype described in this study.

### Pilot phase

A pilot phase was conducted involving eleven adult patients from the Department of Intensive Care at Aarhus University Hospital, Aarhus, Denmark, and Gødstrup Regional Hospital, Herning, Denmark. The pilot phase was inserted as a safety measure to fine-tune procedural analysis and guide iterative improvements to the intervention protocol. The pilot phase also served to optimize deployment and removal procedures to ensure consistent data in the subsequent main study and minimize any patient discomfort in all aspects of the procedures.

### Main feasibility study

The main feasibility study was designed as a prospective single-arm interventional study. Ten patients were included from the Department of Intensive Care at Aarhus University Hospital, Aarhus, Denmark, and Gødstrup Regional Hospital, Herning, Denmark. Patients were included from March 2024 to December 2024. Only patients > 18 years of age, who were tracheostomized for > 7 days, deemed ready for decannulation by intensive care physicians according to local criteria, and were cognitively able to provide informed consent, as well as to understand and perform our tests, were included. Patients were excluded if their tracheostomy wound had been infected or was prone to bleeding.

### Ethical approval and compliance with guidelines

The study was approved by the regional scientific ethics committee (Central Denmark Region, no. 1-10-72-112-22, date of approval: 18/08/2022). All methods were performed in accordance with the relevant national and institutional guidelines and regulations, including the Declaration of Helsinki and the principles of Good Clinical Practice. Individual oral and written informed consent was obtained from all participants prior to inclusion.

## Data collection

Data collection of pulmonary physiology and voice quality has previously been described^16^. An identical approach was taken in this study. In addition, data on wound healing and patient satisfaction were also collected. Data was obtained on predefined assessment points, specifically on the inclusion day after decannulation, where the stoma was open, and after insertion of the sealing disc. Tests were then repeated once daily and after removal of the sealing disc on the last day. The patient satisfaction data were collected on the last day using a dedicated questionnaire.

### Spirometry test

Spirometry was conducted using a Pneumotrac Spirometer (Vitalograph, Buckingham, UK) with Spirotrac version 5 software. Measurements included forced vital capacity (FVC), forced expiratory volume in one second (FEV1), and peak expiratory flow (PEF). The procedure followed the American Thoracic Society/European Respiratory Society guidelines on spirometry^20^.

### Voice quality recordings

In accordance with logopedic consultation, voice recordings were standardized by instructing the patient to count from one to ten. The Apple Voice Memos application on an iPhone 15 (iOS version 17, Apple Inc., Cupertino, CA, USA) was used to record the sound. Voice recordings were assessed qualitatively for hoarseness and breathiness by two blinded assessors and rated with an overall score using a 6-point ordinal scale (0 = inaudible voice; 5 = clear, normal voice)^21^.

### Healing assessment

A qualitative wound healing assessment was done every 24 h. Photographic documentation of the wound healing was obtained using the built-in camera of an iPhone 15 (iOS version 17, Apple Inc., Cupertino, CA, USA). Assessments included residual stoma size, evaluation of excessive granulation tissue, and signs of infection. The outcome measure was the number of days from decannulation until complete wound occlusion around the tail of the sealing disc was observed.

### Questionnaire on patient satisfaction

The patient filled out a short questionnaire consisting of six questions related to the insertion and removal of the sealing disc and the treatment period. Patients rated each question on a 5-point Likert scale. The answers were translated to a numerical score, where higher scores indicated a more favorable response^22^. Depending on the question, the response anchors varied (e.g., 1 = “very poor” to 5 = “excellent” for satisfaction; 1 = “very often” to 5 = “not at all” for mucus and wound secretion symptoms; 1 = “very difficult” to 5 = “very easy” for task difficulty).

### Patient demographics

Patient demographics were extracted from medical records and organized using REDCap electronic data capture tools, hosted by Aarhus University^23,24^. Data included age, sex, comorbidities, cause for hospitalization, days in intensive care unit (ICU), length of in-hospital stay, days of mechanical ventilation, days with tracheostomy, type of tracheostomy, tracheostomy tube size, and readmission to ICU within 30 days.

### Procedure

In the pilot phase, we iteratively optimized the deployment and removal procedures as well as data collection and other handling of the patient. The following describes the finalized protocol of the main feasibility study.

Once the patient was deemed ready for decannulation, the patient was seated in an upright position with extension of the neck, and the tracheostomy tube was removed. Spirometric tests and voice recordings were done with an open stoma. A photo of the tracheostomy wound was also taken. A spray of xylocaine 10% was administered to the wound to minimize any discomfort when placing the sealing disc. The sealing disc was inserted through the stoma, and the external fixation was mounted. As an extra safety feature, the tail of the disc had a hole that was attached to a button on the external fixation. To absorb any wound secretion, a drainage pad (Metalline, REF 23093, Lohmann & Rauscher A/S, Vedbaek, Denmark) was placed around the tracheostomy wound. After inserting the sealing disc, spirometric tests and voice recordings were repeated. As a safety measure, a research team member stayed continuously with the patient throughout the entire study period from deployment to sealing disc removal, and was able to assist should the equipment have to be adjusted or removed. The sealing disc was removed when the wound had healed closely around the “tail” of the disc.

### Statistical analysis

All continuous variables are presented as median with interquartile range (IQR) due to the low sample size. Spirometry results and voice scores were compared pairwise between assessment points (inclusion day: open versus sealed stoma, last day: sealed stoma versus after removal, and finally inclusion day open stoma versus last day after removal) using Wilcoxon signed-rank test. Tests were two-tailed and interpreted at a statistical significance level of 0.05. Statistical analyses were performed using R statistical analysis software (RStudio), version 5.5.4 (R Foundation for Statistical Computing, Vienna, Austria).

## Results

### Pilot phase

Pilot Phase (*n* = 11): Understanding the insertion process of the sealing disc was a key result of the pilot phase. Improper placement and insufficient wound sealing with air leakage were present in eight patients. Five patients experienced discomfort related to the displaced disc. Corrections in the deployment procedure were made, including narrowing the insertion tube, ensuring that the time from tube removal to sealing disc insertion did not exceed three minutes, and deploying the sealing disc deeper into the trachea and then retracting it into the correct position. This ensured proper placement of the sealing disc in the subsequent three patients, and these patients experienced no discomfort. As a safety measure, these patients only had the sealing disc in place for 24 h as they remained in the ICU. No significant air leakage was observed while the sealing disc was in place.

### Main study

Ten patients were included in the feasibility study (Table 1).

**Table 1:** Patient demographics and clinical characteristics of each patient included in the feasibility study.

Pt	Cause of admission	Sex	Age (years)	Tracheostomy technique	Tube inner diameter (mm)	Mechanical ventilation time (days)	Time tracheostomized (days)	Time with sealing disc in place (days)	Decannulation failure	Length of hospital stay (days)
1	Perforated esophagus, postoperative respiratory distress	Female	74	PDT	8.0	18	14	3	No	29
2	Perforated esophagus, postoperative respiratory distress	Male	78	PDT	8.0	32	27	4	Yes	57
3	Thoracic surgery, postoperative acute respiratory distress	Male	73	PDT	8.0	33	24	3	No	45
4	Cardiac arrest, prolonged recovery	Male	64	PDT	8.0	16	5	3	No	52
5	Legionnaires’ disease	Female	49	PDT	7.0 (6.0)*	16	10	3	No	20
6	Cardiac arrest, prolonged recovery	Female	64	PDT	7.0	19	6	3	No	37
7	Cardiac arrest, severe heart failure, chronic need for left ventricular assist device	Male	62	PDT	7.5	30	21	4	No	73
8	Cardiac arrest, prolonged recovery	Male	62	PDT	8.0	43	35	3	No	80
9	Abdominal surgery, intra-abdominal abscess, prolonged infection management	Female	58	PDT	7.0 (6.0)*	36	25	4	No	194
10	Acute respiratory distress syndrome	Male	40	Surgical	8.0	40	23	3	No	48

* Tube downsized.

PDT: percutaneous dilatational tracheostomy.

The included patients were admitted to the ICU due to cardiac arrest (*n* = 4), perforated esophagus with postoperative respiratory distress (*n* = 2), thoracic surgery with postoperative acute respiratory distress (*n* = 1), abdominal surgery with intra-abdominal abscess and prolonged infection management (*n* = 1), acute respiratory distress syndrome (*n* = 1), and Legionnaires’ disease (*n* = 1). The median age was 63 years. Most often, a percutaneous dilatational tracheostomy was performed (9 patients), and only a single patient had a surgical tracheostomy. Patients were mechanically ventilated for a median of 31 days and had a tracheostomy tube for a median of 22 days, with the median tube size 8.0. The median length of hospital stay was 49.5 days. In two patients, spirometry and voice quality recordings were not carried out on the inclusion day before sealing (open stoma) due to downsizing of the tracheostomy tube (Table 1). Based on experience from the pilot phase, narrow tracheostomy tracts (tube size 6.0) required immediate insertion of the sealing disc after decannulation to avoid time delays that could hinder placement.

### Feasibility

The sealing disc deployment was successful and uncomplicated in all ten patients. No patients reported discomfort following deployment. The intratracheal seal appeared airtight in all cases and did not require repositioning of the disc. All patients demonstrated effective airway clearance and were able to expel mucus with no need for tracheal suction. Removal of the sealing disc was accomplished in less than 30 s and was uneventful in all cases, with each disc fully uncoiled and retrieved intact (Supplementary Video S3).

### Patient satisfaction

All ten patients completed a questionnaire about their experience with the tracheostomy sealing disc. As Table 2 shows, patient satisfaction with the sealing disc was generally high across all domains. Median ratings (IQR) for overall experience and comfort during insertion/wear/removal were 5 with an interquartile range of 1 (1), indicating an excellent experience for most patients. Although patients, on one hand, reported perceiving airway mucus relatively often – median score: 3 (3) – on the other hand, they rated the ease of clearing these secretions as excellent – median score: 5 (1). This suggests that, while mucus was present, the impact on patient comfort was limited due to the ease of airway clearance. Importantly, no patient rated their experience as very poor in any category.

**Table 2:** Patient-reported satisfaction and symptom ratings related to use of the sealing disc. Responses were scored on a 5-point Likert scale (1 = very poor/very often/very difficult, 5 = excellent/not at all/very easy). Data is presented as median with interquartile range (IQR).

No	Question	Median score (IQR)
1	How would you rate the comfort of inserting, wearing, and removing the sealing disc?	5 (1)
2	How often did you experience a build-up of mucus in the airways while wearing the sealing disc?	3 (3)
3	How would you rate your ability to relieve mucus from the airways while wearing the sealing disc?	5 (1)
4	How often did you notice fluid or secretion around your tracheostomy wound?	5 (0)
5	How would you rate your ease of speaking while wearing the sealing disc?	5 (0)
6	How would you rate the overall experience of the tracheostomy sealing disc treatment?	5 (1)

IQR: interquartile range.

### Wound healing

**Fig. 5:**
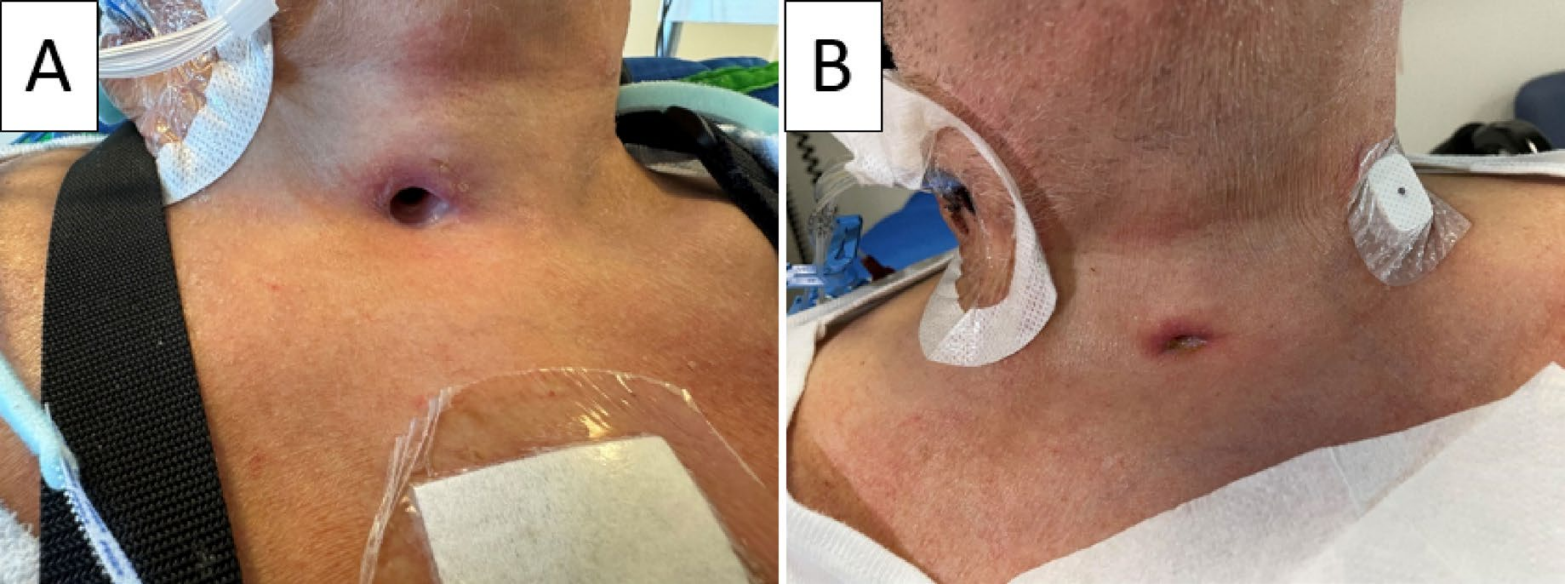
A patient with (**A**) a stoma immediately after decannulation and (**B**) the residual stoma three days later, immediately after sealing disc removal in the same patient. Published with permission of the patient.

Seven of the ten patients experienced complete wound healing around the tail of the sealing disc within three days, and the sealing disc was then removed. The remaining three patients achieved this after four days. Observation of the stoma revealed very limited secretion, and the absorbent dressing was only changed once every 24 h. Upon removal of the sealing disc, a residual stoma measuring approximately 3–5 mm in diameter was present in all cases. Figure 5A illustrates a representative image of the stoma at decannulation, while Fig. 5B shows the residual stoma three days later, immediately after sealing disc removal for the same patient. To further characterize wound closure beyond this timepoint, we managed to re-examine six patients 24–48 h after removal of the sealing disc. At this follow-up, five of the six patients showed complete air-tight closure of the residual stoma, while one patient still exhibited discrete air passage through the tracheostomy. This patient is described in detail in the section on decannulation failure, as insufficient airway clearance in this case ultimately necessitated reintubation and ICU readmission.

### Spirometry

The results of the spirometry are seen in Table 3.

**Table 3:** Spirometry results presented as median with interquartile range (IQR) at predefined assessment timepoints.

Variable	Number of patients	FVC (mL)	FEV_1_ (mL)	PEF (L/min)
		Median (IQR)	Median (IQR)	Median (IQR)
Inclusion day: Open stoma	8	897 (503–1515)	757 (490–1143)	101 (79–154)
Inclusion day: Sealed stoma	10	1523 (1107–2509)	1257([740–1675)	140 (85–218)
Day 1: Sealed stoma	10	1653 (1436–2409)	1008 (750–1583)	93 (88–162)
Day 2: Sealed stoma	10	1863 (1341–2295)	1245 (805–1611)	150 (83–183)
Day 3: Sealed stoma	10	1698 (1225–2499)	1178 (670–1788)	121 (80–203)
Day 4: Sealed stoma	3	1545 (1258–1720)	920 (753–1193)	137 (104–147)
After removal	10	1415 (1194–2398)	1065 (845–1833)	138 (95–191)

FEV_1_: forced expiratory volume in one second; FVC: forced vital capacity; IQR: interquartile range; PEF: peak expiratory flow.

As the patient characteristics of Table 1 show, downsizing of the tracheostomy tube to a size 6 was done in two patients prior to inclusion. In these two cases, baseline recordings of “Inclusion day Open stoma” were not carried out. This leaves eight patients eligible for paired analysis of spirometric tests before and after insertion of the sealing disc. As presented in Table 4, comparison of “Inclusion day Open stoma” versus “Inclusion day Sealed stoma” demonstrated increases in FVC, FEV₁, and PEF following insertion of the sealing disc. The same table further shows that after disc removal, spirometry with the small residual stoma yielded higher values than with the open stoma on the day of inclusion. Spirometric values with the residual stoma (After removal) were even comparable to those obtained with the stoma sealed, with only FVC showing a significant decrease, indicating that the residual stoma had only a limited impact on lung function tests.

**Table 4:** Spirometry outcomes presented as median with interquartile range (IQR) at predefined assessment timepoints for the eight patients eligible for comparative analysis (inclusion day: open stoma, inclusion day: sealed stoma, last day: sealed stoma, last day: after removal).

Variable	Assessment timepoints				*P*-value		
	Inclusion day		Last day		Inclusion day	Inclusion day	Last day
	Open stoma	Sealed stoma	Sealed stoma	After removal	Open vs. Sealed	Open vs. After removal	Sealed vs. After removal
FVC (mL)	897 (503–1515)	1523 (1300–2354)	1748 (1496–2499)	1415 (1241–2574)	0.014	0.021	0.032
FEV_1_ (mL)	757 (490–1143)	1257 (820–1638)	1338 (929–1788)	1065 (855–1658)	0.014	0.030	0.114
PEF (L/min)	101 (79–154)	140 (87–218)	147 (110–203)	138 (96–188)	0.294	0.011	1.000

FEV_1_: forced expiratory volume in one second; FVC: forced vital capacity; PEF: peak expiratory flow.

### Voice quality

The voice quality scores are seen in Table 5.

**Table 5:** Voice quality scores (6-point equal-appearing-interval scale; 0 = inaudible voice; 5 = clear, normal voice) across assessment timepoints (inclusion day: open and sealed stoma, day 1–4: sealed stoma, and after disc removal with residual stoma) presented as median with interquartile range.

Assessment timepoint	Number of patients	Median score (IQR)
Inclusion day: Open stoma	8	1 (1)
Inclusion day: Sealed stoma	10	4 (0)
Day 1: Sealed stoma	7	5 (0)
Day 2: Sealed stoma	8	5 (0)
Day 3: Sealed stoma	9	5 (0)
Day 4: Sealed stoma	3	5 (0)
After removal	10	4 (1)

Just as with spirometry, two patients had no baseline recordings of from the day of inclusion and on “Sealed stoma Days 1–3”, 6 recordings were excluded from scoring because of excessive noise and inadequate recording quality. This leaves eight patients eligible for paired analysis of voice quality scores with open and sealed stoma on the inclusion day, and sealed and residual stoma after disc removal, on their last day. When comparing median voice quality scores (IQR) for open stoma immediately after decannulation to scores with the stoma sealed at the inclusion day, the voice quality significantly increased: 1 (1) vs. 4 (0), *p* = 0.014. Also, when comparing scores for open stoma immediately after decannulation on the day of inclusion to residual stoma after removal of the sealing disc on the last day, the voice quality increased: 1 (1) vs. 4 (1), *p* = 0.013.

### Decannulation failure

One patient experienced insufficient airway clearance five days post-decannulation, resulting in decannulation failure with intubation and renewed mechanical ventilation for two days. This happened 24 h after the sealing disc was removed. The patient received CPAP three times daily since the day of decannulation. The tracheostomy wound was not fully healed when the patient was readmitted to the ICU.

## Discussion

This first-in-man feasibility study demonstrates both the feasibility and indicates the clinical potential for intratracheal sealing of the tracheostomy following decannulation. The pilot phase allowed our research team to become proficient in device handling and optimizing the procedure, which resulted in a smooth and well-tolerated deployment, wear, and removal in the main study. The sealing disc provided an airtight seal during speaking and coughing, effectively preventing air leakage frequently observed with conventional external dressings^25–27^. This intraluminal closure supported key physiological functions during the critical post-decannulation phase, including redirection of airflow through the upper airway to restore subglottic pressure necessary for coughing and speaking^28,29^. Objective spirometry data showed consistent improvements in ventilatory parameters across the intervention period, while voice quality also improved, reflecting enhanced expiratory flow dynamics.

Furthermore, healing of the tracheostomy wound was present around the tail within 3–4 days, while a small residual stoma was evident after removal of the sealing disc in all patients. Five out of six patients achieved airtight closure of the residual tracheostomy within 24–48 h, corresponding to full closure after four to six days in total. This is faster than typically observed with passive occlusive dressings, where the median time from decannulation to full closure is 7^3^. These findings suggest that the sealing disc not only preserves airway integrity while in situ but may also accelerate wound healing once removed. However, these results are indicative and need to be investigated further. Importantly, patient satisfaction regarding comfort, ease of speech, and wound secretion was high and indicated satisfactory mucus clearance when needed.

Notably, one patient required ICU readmission after sealing disc removal due to secretion stagnation. While this may represent an incidental finding, it is consistent with the observation that cough strength and secretion clearance were adequate while the disc was in place, but less effective after removal when a small residual opening remained. This case suggests that maintaining airway patency in the early post-decannulation period is crucial, particularly in fragile patients with marginal respiratory reserve.

Our findings align with emerging recognition that airtight closure of the tracheostomy site after decannulation is clinically important. Other authors have reported similar findings and proposed alternative sealing methods to prevent air leakage and maintain subglottic pressure during early healing^25^. This convergence of findings across different techniques suggests that ensuring airtightness may be a realizable principle for optimizing post-decannulation recovery, regardless of the specific device or approach used.

In addition, our work addresses a broader gap in post-decannulation care. A Danish study highlighted that follow-up of tracheostomized patients is often inadequate, particularly when decannulation occurs outside the ICU setting^30^. Traditional tracheostomy wound care after decannulation requires intensive maintenance; if inadequate in general wards, residual airway compromise may go undetected, delaying recovery and increasing the risk of complications – in particular relapse to renewed mechanical ventilation therapy – a stressful experience for the patient and an expensive treatment for the health care system^12,17,19,31^.

Future refinement should focus on reducing the interval between sealing disc removal and full skin closure – potentially by making the disc thinner to allow extended use – and on determining the optimal sealing duration and target patient subgroups. In addition, future research should investigate whether the sealing method can facilitate earlier and safer decannulation by supporting cough strength and airway integrity, thereby reducing the risk of re-cannulation and associated healthcare costs^32^.

## Limitations

This study has several limitations inherent to its feasibility design. The small sample size limits the generalizability of the findings and prevents advanced statistical analysis. Feasibility was assessed in a controlled clinical setting by clinicians familiar with the device, and the findings may therefore not directly reflect usability when applied by less experienced users in routine clinical practice. The spirometric comparison against an open stoma represents a worst-case physiological condition rather than standard post-decannulation care. In routine practice, the dressings placed over the stoma will partially mitigate air leakage with varying effects. Therefore, the observed magnitude of spirometric improvement should be interpreted with caution. The absence of a control group restricts direct comparisons with standard post-decannulation care. The dedicated questionnaire was specifically designed to uncover patient aspects regarding this new treatment method. The questionnaire assessing patient satisfaction was not validated prior to application, limiting comparability and interpretability of absolute scores. Additionally, voice quality and wound healing were assessed using qualitative or semi-quantitative methods, which may introduce observer bias. In order to limit the number of additional procedures and the degree of invasiveness in this first-in-human study, we refrained from including endoscopic follow-up of the intratracheal aspect during the healing period. The downside of that approach evidently precludes us from performing a detailed assessment of mucosal changes intratracheally, such as granulation tissue formation, etc. In a planned larger prospective randomized study, we consider expanding the study design with endoscopic follow-up. Finally, only patients able to provide informed consent and physically capable of completing spirometry and voice assessments were included, which may introduce a selection bias toward less severely affected individuals.

## Conclusion

This first-in-human feasibility study provides promising indications that intratracheal sealing using a third-generation tracheostomy sealing disc is feasible and well tolerated, with no device-related adverse events observed during short-term post-decannulation follow-up. The sealing disc enabled effective airway sealing, restored pulmonary function and voice quality, and was associated with high patient satisfaction and favorable wound healing outcomes. These findings support the potential clinical value of intratracheal sealing in preserving airway integrity and facilitating recovery during the critical post-decannulation phase. Further studies are warranted to confirm these results in larger, controlled cohorts and to explore whether this approach can promote earlier and safer decannulation and eventually improve pulmonary rehabilitation and patient recovery compared to conventional post-decannulation tracheostomy care.

## Supplementary Information

Below is the link to the electronic supplementary material.

Supplementary Material 1

Supplementary Material 2

Supplementary Material 3

Supplementary Material 4

## Data Availability

The datasets generated and analyzed during the current study are available from the corresponding author on reasonable request.
