# Supplementary material for: Clinical feasibility of intratracheal tracheostomy sealing using a novel sealing disc prototype
Source: Sci Rep. 2026 Mar 24;16:14800. doi: 10.1038/s41598-026-41209-8 (PMC13168437; doi:10.1038/s41598-026-41209-8)
Supplement: Supplementary file 4 — Supplementary Material 4 [file 41598_2026_41209_MOESM4_ESM.docx]

## Supplementary Video Legends

Supplementary Video S1: Video showing the insertion process of the sealing disc. Audio removed to ensure patient anonymity and confidentiality. Published with permission of the patient.

Supplementary Video S2: Bronchoscopic view of the removal process of the sealing disc in a preclinical porcine model.

Supplementary Video S3: Video showing the removal process of the sealing disc. Audio removed to ensure patient anonymity and confidentiality. Published with permission of the patient.
